# Modeling sarcoplasmic reticulum Ca^2+^ in rat cardiomyocytes

**DOI:** 10.52601/bpr.2024.240012

**Published:** 2024-10-31

**Authors:** Yutong Su, Yongshen Liang, Menghao Xu, Beibei Gao, Siyuan Zhang, Eric Yang, Shuai Yin, Da Li, Zhangqin Huang, Wenjun Xie

**Affiliations:** 1 Beijing Engineering Research Center for IoT Software and Systems, Beijing University of Technology, Beijing 100124, China; 2 Department of Cardiology, First Affiliated Hospital of Xi'an Jiaotong University, Xi’an 710061, China

**Keywords:** Numerical model, Sarcoplasmic reticulum, Calcium, Rat cardiomyocytes, Fluorescence dye

## Abstract

The sarcoplasmic reticulum (SR) primarily serves as the intracellular Ca^2+^ store in cardiac myocytes, mediating cellular function under cardiac physiology and diseases. However, the properties of cardiac SR Ca^2+^ have not yet been fully determined, particularly in rats and mice, which are the most commonly used experimental species in studies on cardiac physiology and diseases. Here, we developed a spatially detailed numerical model to deduce Ca^2+^ movements inside the junctional SR (jSR) cisternae of rat cardiomyocytes. Our model accurately reproduced the jSR Ca^2+^ kinetics of local and global SR Ca^2+^ releases reported in a recent experimental study. With this model, we revealed that jSR Ca^2+^ kinetics was mostly determined by the total release flux via type 2 ryanodine receptor (RyR2) channels but not by RyR2 positioning. Although Ca^2+^ diffusion in global SR was previously reported to be slow, our simulation demonstrated that Ca^2+^ diffused very quickly inside local jSR cisternae and the decrease in the diffusion coefficient resulted in a significant reduction of jSR Ca^2+^ depletion amplitude. Intracellular Ca^2+^ was typically experimentally detected with fluorescence dye. Our simulation revealed that when the dynamical characteristics of fluorescence dye exerted a minimal effect on actual Ca^2+^ mobility inside jSR, the reaction rate of the dye with Ca^2+^ could significantly affect apparent jSR Ca^2+^ kinetics. Therefore, loading a chemical fluorescence dye with fast kinetics, such as Fluo-5N, into SR is important for Ca^2+^ measurement inside SR. Overall, our model provides new insights into deciphering Ca^2+^ handling inside nanoscopic jSR cisternae in rat cardiomyocytes.

## INTRODUCTION

In cardiac myocytes, the sarcoplasmic reticulum (SR) primarily serves as the intracellular Ca^2+^ store that provides most contractile Ca^2+^ to myofilaments (Bers 2002; Eisner *et al*. 2017). Structurally, SR is a continuous membrane-bound organelle that extends across the cytosolic space with interconnected nanotubules, *i*.*e*., free SR (fSR), and flat cisterns, *i*.*e*., junctional SR (jSR), which form junctions with transverse tubules (TTs) at the level of the sarcomeric Z lines (Brochet *et al*. 2005). Ca^2+^ release from cardiac SR is gated by type 2 ryanodine receptor (RyR2), which is the main Ca^2+^ release channel located at the jSR membrane inside SR–TT junctions, via the Ca^2+^-induced Ca^2+^ release mechanism (Bers 2002). This mechanism generates multiscale and multimodal cytosolic Ca^2+^ signals, including quarky Ca^2+^ release, Ca^2+^ sparks, Ca^2+^ transients, and Ca^2+^ waves, in cardiomyocytes (Cheng *et al*. 2008; Brochet *et al*. 2011). SR Ca^2+^ dynamics is currently recognized to play a critical role in the regulation of RyR2 gating, controlling cytosolic Ca^2+^ pattern and cellular Ca^2+^ homeostasis (Eisner *et al*. 2017; Jones *et* *al*. 2017; Zhang *et al.* 2021). Therefore, further deciphering SR Ca^2+^ dynamics in cardiac physiology and diseases is informative.

With the development of a protocol that favors the SR retention of low-affinity Ca^2+^ indicators Fluo-5N in 2005, Ca^2+^ blinks, which are local SR Ca^2+^ events that accompany Ca^2+^ sparks, were first experimentally observed in rabbit cardiomyocytes (Brochet *et al*. 2005). Following this report, a few studies have revealed cardiac SR Ca^2+^ kinetics and its regulation of cellular Ca^2+^ homeostasis by interplaying with cytosolic Ca^2+^ events (Zima *et al*. 2008; Brochet *et al*. 2011, 2012; Domeier *et al*. 2012; Greensmith *et al*. 2014; Wang *et al*. 2014; Bovo *et al*. 2015). To date, however, such a strategy for imaging SR Ca^2+^ has failed to achieve widespread application in cardiomyocytes from species other than rabbit and sheep, limiting our knowledge about cardiac SR Ca^2+^ to these species. By expressing SR-targeted carboxylesterase via adenoviruses, Lu *et al*. successfully imaged SR Ca^2+^ in intact cardiomyocytes from rats and mice (Lu *et al*. 2020), small rodents that are most commonly used in cardiovascular research, providing most of our knowledge about cardiac Ca^2+^ signaling, particularly in heart diseases. Although quantitative measurements of cytosolic Ca^2+^ dynamics in cardiomyocytes have demonstrated considerable differences among species (Bers 2002; Eisner *et al*. 2017), further extending our knowledge about SR Ca^2+^ signals to murine cardiomyocytes is urgent.

Complementing experimental studies, computational modeling has further demonstrated nanoscale details for Ca^2+^ mobilization, which is experimentally limited by optical resolution. Although most models have been developed for cytosolic Ca^2+^ kinetics, several models have also simulated SR Ca^2+^ kinetics (Sobie *et al*. 2005; Zima *et al*. 2008; Cannell *et al*. 2013; Li *et al*. 2017). In particular, Cannell *et al*. developed a model with considerable details in the dyad and SR network; this model reproduced most of the spatiotemporal characteristics of Ca^2+^ sparks/blinks and their termination mechanism (Cannell *et al*. 2013). Li *et al*. developed a spatially detailed model to describe Ca^2+^ movements inside the jSR of cardiomyocytes (Li *et al*. 2017). However, on the basis of Ca^2+^ blinks in rabbit cardiomyocytes, these previous simulations of SR Ca^2+^ kinetics did not agree well with a recent experimental study on rat cardiomyocytes (Lu *et al*. 2020).

In the current work, we developed a spatially detailed numerical model to deduce Ca^2+^ movements inside the jSR cisternae of rat cardiomyocytes. Our model not only agreed well with the experimental Ca^2+^ kinetics inside jSR but also revealed the influences of jSR Ca^2+^ diffusion and fluorescence dye characteristics on apparent jSR Ca^2+^ kinetics.

## RESULTS

**Figure 1:**
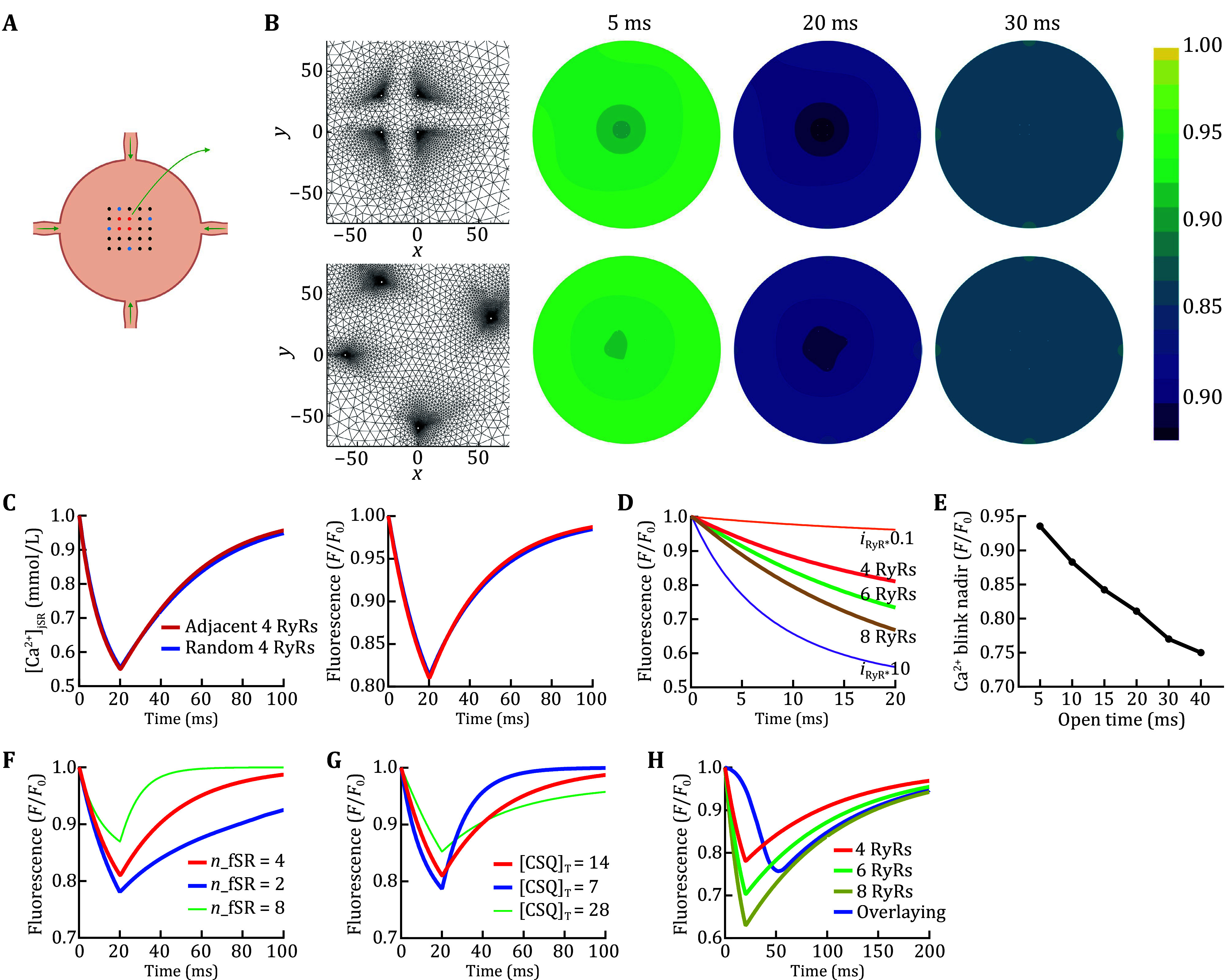
Simulations of Ca^2+^ kinetics inside the jSR of rat cardiomyocytes. **A** Schematic of jSR and the connected fSR network. Dots represent RyR2 channels. **B** The unstructured grids near the open RyR2 channels and fluorescence contour at the indicated time points. Top, four adjacent RyR2 channels that correspond to the red dots in panel A. Bottom, four random RyR2 channels that correspond to the blue dots in Panel A. **C** The simulated time course curves of Ca^2+^ concentration (left) and normalized Fluo-5N fluorescence (right) in the jSR of rat cardiomyocytes in response to the four adjacent (red) or random (blue) RyR2 channels. The curves represent the average Ca^2+^ or CaF concentration in the whole jSR. **D** The simulated curves of Fluo-5N fluorescence changes in response to the 20 ms opening of the indicated number of open RyR2 channels, or four open RyR2 channels with *i*_*RyR*_ adding/reducing to 10 folds of the original value. **E** The modeled Ca^2+^ blink nadir with different opening times of four RyR2 channels. **F**,**G** The modeled Ca^2+^ blink curve in response to the 20 ms opening of four adjacent RyR2 channels with different numbers of refilling fSR tubules or CSQ concentrations. **H** The simulated curves for Ca^2+^ depletion transient inside the jSR of rat cardiomyocytes by overlaying 100 jSR CaF traces from 4–8 open RyR2 channels with 1/3 fSR refilling flux and 0–50 ms Gaussian-distributed latency

In rat cardiomyocytes, a typical Ca^2+^ spark event corresponds to Ca^2+^ release with a peak flux of ~2.5 pA (Wang *et al*. 2004) from the jSR cisterna. This value is four times that of a single RyR current (~0.6 pA) under physiological conditions (Mejia-Alvarez *et al*. 1999). The average lasting time for SR Ca^2+^ release is ~20 ms during a Ca^2+^ spark (Lu *et al*. 2020). We then modeled the accompanying Ca^2+^ event inside jSR, *i*.*e*., Ca^2+^ blink, with an opening of 20 ms for four adjacent RyR2 channels located at (0, 0), (0, 30), (30, 30), and (30, 0) in the jSR cisterna (Fig. 1B, top). As shown in Fig. 1C, the modeling results of Ca^2+^ release through the four channels agree with the experimental Ca^2+^ blink kinetics of rat cardiomyocytes (Lu *et al*. 2020) with an amplitude (in Δ*F*/*F*_0_) of 0.185 (model) versus 0.190 (experiment) and a full duration at half maximum (FDHM; in ms) of 34.1 (model) versus 32.8 (experiment). The depletion of free Ca^2+^ in jSR cisternae reaches up to 45%, which is ~2.4 times of the apparent depletion indicated by Fluo-5N fluorescence intensity in a Ca^2+^ spark/blink pair (Fig. 1C).

Although several previous studies have reported the interplay between adjacent RyR2 channels in the same CRU (Xie *et al*. 2010), the locations of open RyR2 channels in a Ca^2+^ spark/blink pair remain unsolved (Cheng *et al*. 2008). We then assessed the effect of open RyR2 positioning on jSR Ca^2+^ kinetics by replacing the adjacent RyR2 opening with four randomly located channels (Fig. 1B, bottom). Such alteration exerted nearly no effect on the modeled Ca^2+^ blink kinetics (Fig. 1C). Meanwhile, the alteration of the total releasing flux, which can result from changing the number of open RyR2 channels, or *i*_RyR_, or the open time of RyR2, evidently affect the nadir of the modeled Ca^2+^ blink (Figs. 1D and 1E). Previous studies have indicated that refilling flux from fSR tubules and the buffering capacity of CSQ are important in Ca^2+^ blink kinetics (Li *et al*. 2017). Similarly, our simulations demonstrated that changes in refilling fSR tubule number (referred to as the refilling flux hereafter) and CSQ concentrations could affect Ca^2+^ blink nadir and recovery time (Figs. 1F and 1G).

Ca^2+^ transient is the summation of Ca^2+^ spark/blink throughout a cell. It involves more RyR2 openings at each CRU (Cheng *et al*. 2008) and exhibits a triple recovery time for cytosolic and jSR [Ca^2+^] compared with a single spark/blink pair (Lu *et al*. 2020). We modeled jSR Ca^2+^ depletion transient kinetics by overlaying 100 modeled jSR CaF traces from 4–8 open RyR2 channels with 1/3 fSR refilling flux and 0–50 ms Gaussian-distributed latency (Song *et al*. 2001). As shown in Fig. 1H, the modeled jSR Ca^2+^ depletion transient exhibited 24.3% depletion of CaF fluorescence, time to nadir of 48.5 ms, and half recovery time of 69.5 ms, which are extremely close to the experimental characteristics of jSR Ca^2+^ depletion transients (Lu *et al*. 2020).

**Figure 2:**
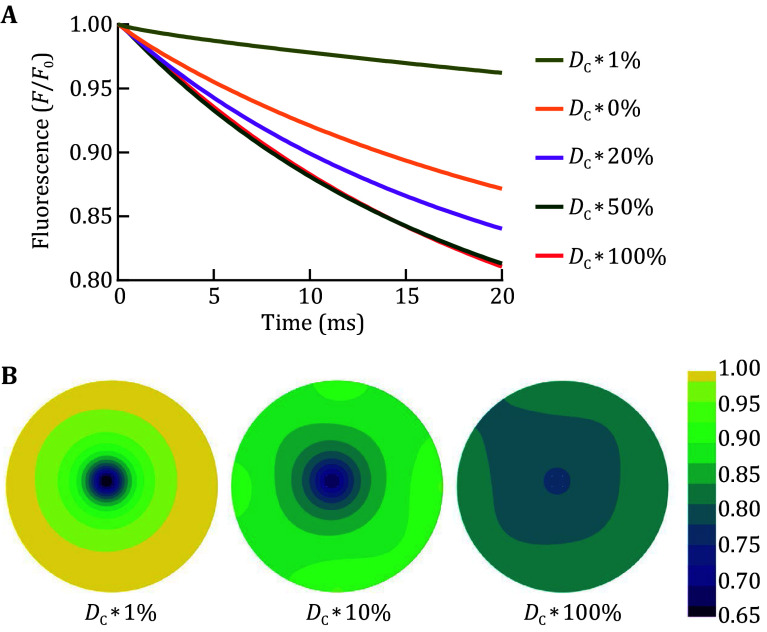
Effect of the reduction of the Ca^2+^ diffusion coefficient inside jSR on modeled Ca^2+^ blink amplitude. **A** The simulated time course curves of Fluo-5N fluorescence in response to the 20 ms opening of four RyR2 channels with different Ca^2+^ diffusion coefficients. **B** The fluorescence contour of the indicated Ca^2+^ diffusion coefficients at a time point of 20 ms

Previous experimental studies have reported a relatively slow mobility of Ca^2+^ inside global SR (with one or two orders of magnitude lower apparent diffusion coefficient than that in cytosolic space) (Swietach *et al*. 2008), which is largely limited by the extremely thin fSR network. The manner in which Ca^2+^ diffuses inside the relatively wider jSR space remains unknown. While reproducing the experiment on Ca^2+^ blink dynamics by using the same Ca^2+^ diffusion coefficient inside jSR as that in cytosolic space, we tested the effect of the reduced Ca^2+^ diffusion coefficient inside jSR on Ca^2+^ blink kinetics. The decrease in Ca^2+^ diffusion coefficient to 20%, 10%, or 1% resulted in decreases of the modeled Ca^2+^ blink amplitude by about 16%, 32%, or 80%, respectively (Fig. 2A). The contour displayed steeper gradients around the open RyR2 channels with a reduction in the Ca^2+^ diffusion coefficient (Fig. 2B).

**Figure 3:**
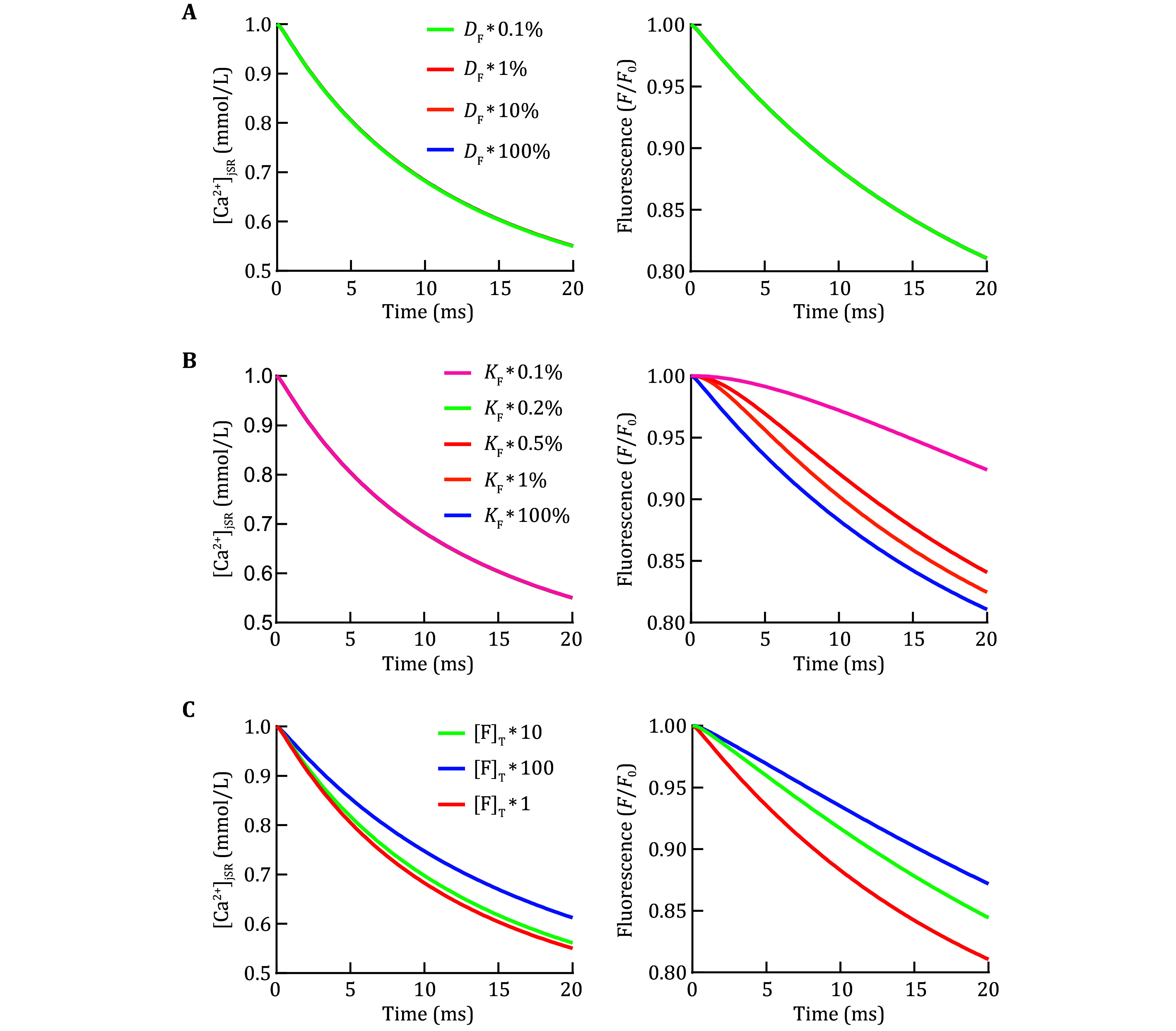
Effect of the characteristics of fluorescence dye on the modeled Ca^2+^ blink amplitude. The simulated time course curves of Ca^2+^ concentration (left) and normalized Fluo-5N fluorescence (right) in the jSR of rat cardiomyocytes with different CaF diffusion coefficients (**A**), reaction rates between the dye and Ca^2+^ (**B**), or total Fluo-5N concentrations (**C**)

Experimental jSR Ca^2+^ was visually measured with a fluorescence indicator; hence, we also assessed its influences on Ca^2+^ and CaF kinetics. As displayed in Fig. 3A, the diffusion coefficient of the fluorescence indicator exerted no effect on Ca^2+^ and fluorescent signals. The association and dissociation constants determine the reaction rate of fluorescent dye with Ca^2+^. A reduction in the association and dissociation constants did not affect jSR Ca^2+^ kinetics, but considerably decreased the sensitivity of fluorescent signals (Fig. 3B). Notably, the total concentration of the Ca^2+^ indicator should be controlled to an appropriate range, because it also serves as a Ca^2+^ buffer and can considerably affect Ca^2+^ and fluorescent signals inside jSR (Fig. 3C).

## DISCUSSION

SR functions as an intracellular Ca^2+^ store in cardiomyocytes (Bers 2002; Eisner *et al*. 2017). However, the properties of cardiac SR Ca^2+^ have not been fully determined, particularly in small rodents, such as rats and mice, which are the most commonly used experimental species in studies on cardiac physiology and diseases (Lu *et al*. 2020). Here, on the basis of a recent experimental study (Lu *et al*. 2020), we developed a spatially detailed numerical model to deduce Ca^2+^ movements inside the jSR cisternae of rat cardiomyocytes. With this model, we reproduced local SR Ca^2+^ event, Ca^2+^ blink, and global Ca^2+^ depletion transient in rat cardiomyocytes.

The number of RyR2 channels that open during the Ca^2+^ spark remains controversial. An early study revealed that Ca^2+^ sparks in rat ventricular myocytes exhibited quantized Ca^2+^ release flux with a quantal unit of 1.24 pA and a peak histogram of quantal number at 2 (Wang *et al*. 2004). Thus, a typical Ca^2+^ spark corresponds to a total SR releasing flux of ~2.5 pA. We modeled four RyR2 openings during a typical local SR Ca^2+^ release event, because a single RyR2 current was measured as ~0.6 pA in the bilayer lipid membrane (Mejia-Alvarez *et al*. 1999). Our simulated Ca^2+^ blink kinetics agreed with the recently reported experimental results (Lu *et al*. 2020). Considering that *in vivo* single RyR2 current might differ from this value (0.6 pA), our simulations further revealed that the total release flux, but not the number or positioning of open RyR channels, determined jSR Ca^2+^ kinetics in rat cardiomyocytes.

Compared with the open space of cytosol, the intraluminal space of thin fSR tubules limits Ca^2+^ mobilization in the SR network, and thus, a slow diffusion of Ca^2+^ in global SR is identified (Swietach *et al*. 2008). In the current study, we determined that a reduction in the jSR Ca^2+^ diffusion coefficient resulted in an evidently decreased amplitude of the simulated Ca^2+^ blink, while simulation with Ca^2+^ mobilization in jSR that was as fast as cytosol concurred with the experimental results. Such fast mobilization of Ca^2+^ inside jSR also accounts for the unimportance of open RyR2 positioning.

When the chemical fluorescence dye is extremely difficult to load into SR in myocytes, the genetically encoded Ca^2+^ indicators (GECIs) provide an alternative strategy. Several low-affinity GECIs located in SR lumen were developed previously (Kasai *et al*. 2004; Jimenez-Moreno *et al*. 2010). However, these GECIs can only detect global Ca^2+^ changes, but fail in detecting fast local SR Ca^2+^ events, such as blinks in cardiomyocytes. These GECIs typically have considerably slower kinetics than synthetic chemical Ca^2+^ indicators (Jimenez-Moreno *et* *al*. 2010). In the current study, our simulation suggested that the reaction rate of Ca^2+^ and dye remarkably affected the sensitivity of detecting apparent Ca^2+^ changes in jSR, while the real Ca^2+^ kinetics remained unaltered. Thus, the development of a straegy for loading a chemical Ca^2+^ dye into cardiac SR is crucial for research on SR Ca^2+^ signals (Lu *et al*. 2020). A prior simulation study described heavy underestimation of the real local SR Ca^2+^ depletion (~80%) by Fluo-5N signals (~0.2 Δ*F*/*F*_0_) due to optical blurring and noise (Kong *et al*. 2013). However, this result disagreed with the recent experimental study on rat cardiomyocytes that displayed a considerably smaller apparent amplitude of SR Ca^2+^ during Ca^2+^ sparks (~0.19 Δ*F*/*F*_0_) and transients (~0.26 Δ*F*/*F*_0_) compared with caffeine-induced SR Ca^2+^ depletion (~0.67 Δ*F*/*F*_0_) (Lu *et al*. 2020). Thus, determining the extent to which optical blurring and noise can affect Fluo-5N signals in the SR of rat cardiomyocytes calls for further investigation.

Our present model has several limitations. Previous experimental and modeling studies have revealed the importance of the dyad space between jSR and TTs. As a relatively closed space between jSR and open cytosol, dyad Ca^2+^ kinetics is highly distinct from them (Cannell *et al*. 2013; Shang *et al*. 2014). While most models of Ca^2+^ sparks could not reproduce experimental spatial characteristics, *e*.*g*., full width at half maximum (FWHM), modeling with detailed spatial geometry of dyad has solved the problem of FWHM (Cannell *et al*. 2013). Dyad Ca^2+^ concentration is also crucial for RyR2 gating, release flux, and the resultant jSR Ca^2+^ kinetics. For cytosolic modeling, our present model used a previous model without precisely describing the dyad compartment and RyR2 gating. During an SR Ca^2+^ release event, RyR2 channels open and close stochastically, and the diversity of the number and opening time of RyR2 produces SR Ca^2+^ releasing events with changed amplitude and mass (Brochet *et al*. 2011). In some cases, the random switch of RyR2 channels can lead to multiple releases of SR Ca^2+^, resulting in prolonged Ca^2+^ release events (Brochet *et al*. 2011). In the present study, we used a simple fixed open time but not a random switch mode for RyR2 channels. Therefore, while reproducing the average Ca^2+^ blink kinetics, our model is limited from further deciphering jSR and cytosolic Ca^2+^ movements.

The properties of cardiac sarcoplasmic reticulum (SR) Ca^2+^ have not yet been fully determined experimentally and theoretically in small rodents, such as rats and mice, which are the most commonly used experimental species in studies on cardiac physiology and diseases. In the present work, we have demonstrated a simple numerical model that provides an extremely useful investigative tool for understanding SR Ca^2+^ handling in rat cardiomyocytes, and our numerical model provides new insights into the understanding of Ca^2+^ dynamics in the SR of rat cardiomyocytes.

## METHODS

### Model geometry

The geometry of jSR cisternae in murine cardiomyocytes is morphologically and structurally similar to that of rabbits (Brochet *et al*. 2005, Rog-Zielinska *et al*. 2021). Thus, we simplified Ca^2+^ diffusion inside a flat pancake-shaped jSR with a diameter of 600 nm and an altitude 30 nm as 2D movements in a circle with four connecting fSR tubules of 30 nm diameter along its periphery (Fig. 1A). The Ca^2+^ release unit (CRU) in jSR is composed of a square RyR2 array with a size of 5 × 5 and an interval of 30 nm between adjacent channels (Fig. 1A).

### Reaction/diffusion equations

When a few RyR2 channels in CRU suddenly open, considerable [Ca^2+^] gradients between both sides of the channels drive a rapid and substantial outflow of Ca^2+^ from jSR to the cytosol of cardiac myocytes, forming a local or global Ca^2+^ release event. Intra-jSR [Ca^2+^] is determined by the following fluxes: local diffusion that follows Fick’s law, binding of Ca^2+^ to buffers in jSR cisternae, efflux through RyR2 channels, and influx via connecting fSR tubules. To mimic experimental Ca^2+^ signals, a fluorescent dye (Fluo-5N) is also introduced into our model. It binds Ca^2+^ to form the fluorescence compound (CaF), indicating alterations in free [Ca^2+^]. Thus, the governing equations for jSR Ca^2+^ can be written as

1 \begin{equation*}\begin{split} 
\frac{\partial [{\mathrm{C}\mathrm{a}}^{2+}]}{\partial t}=\;&{D}_{\mathrm{C}}\times \left(\frac{{\partial }^{2}[{\mathrm{C}\mathrm{a}}^{2+}]}{{\partial x}^{2}}+\frac{{\partial }^{2}[{\mathrm{C}\mathrm{a}}^{2+}]}{{\partial y}^{2}}\right)\\&
+{J}_{\mathrm{b}\mathrm{u}\mathrm{f}}+{J}_{\mathrm{d}\mathrm{y}\mathrm{e}}+{J}_{\mathrm{R}\mathrm{y}\mathrm{R}}+{J}_{\mathrm{r}\mathrm{e}\mathrm{f}} ,
\end{split}\end{equation*}

2 $ \frac{\partial \left[\mathrm{C}\mathrm{a}\mathrm{F}\right]}{\partial t}={D}_{\mathrm{F}}\times \left(\frac{{\partial }^{2}\left[\mathrm{C}\mathrm{a}\mathrm{F}\right]}{{\partial x}^{2}}+\frac{{\partial }^{2}\left[\mathrm{C}\mathrm{a}\mathrm{F}\right]}{{\partial y}^{2}}\right)-{J}_{\mathrm{d}\mathrm{y}\mathrm{e}} , $

where *D*_C_ and *D*_F_ denote the diffusion coefficients of Ca^2+^ and CaF with values of 350 µm^2^/s and 20 µm^2^/s, respectively (Smith *et al*. 1998).

The flux for calsequestrin (CSQ), the primary Ca^2+^ buffer in jSR, is given by

3 \begin{equation*}\begin{split} 
{J}_{\mathrm{b}\mathrm{u}\mathrm{f}}=& -{k}_{\mathrm{C}\mathrm{S}\mathrm{Q}}^+\times [{\mathrm{C}\mathrm{a}}^{2+}]\times \left({[\mathrm{C}\mathrm{S}\mathrm{Q}]}_{T}-[\mathrm{C}\mathrm{a}\mathrm{C}\mathrm{S}\mathrm{Q}]\right)+{k}_{\mathrm{C}\mathrm{S}\mathrm{Q}}^-\\&
\times [\mathrm{C}\mathrm{a}\mathrm{C}\mathrm{S}\mathrm{Q}] ,
\end{split}\end{equation*}

4 $ \frac{\partial \left[\mathrm{C}\mathrm{a}\mathrm{C}\mathrm{S}\mathrm{Q}\right]}{\partial t}=-{J}_{\mathrm{b}\mathrm{u}\mathrm{f}} , $

where [CaCSQ] represents the concentration of Ca^2+^ that binds to CSQ. The three constants, [CSQ]_*T*_, $ {k}_{\mathrm{C}\mathrm{S}\mathrm{Q}}^{+} $, and $ {k}_{\mathrm{C}\mathrm{S}\mathrm{Q}}^{-} $, denote the total concentration of CSQ (14 mmol/L), and the association (100.0 (µnol/L)^−1^·s^−1^) and dissociation (60,000 s^−1^) parameters in the reaction of CSQ and Ca^2+^, respectively (Li *et al*. 2017).

The fluxes for the indicator dye take the form of

5 $ {J}_{\mathrm{d}\mathrm{y}\mathrm{e}}=-{k}_{\mathrm{F}}^+\times [{\mathrm{C}\mathrm{a}}^{2+}]\times \left({[\mathrm{F}]}_{\mathrm{T}}-[\mathrm{C}\mathrm{a}\mathrm{F}]\right)+{k}_{\mathrm{F}}^-\times [\mathrm{C}\mathrm{a}\mathrm{F}] , $

where [F]_T_, $ {k}_{\mathrm{F}}^{+} $, and $ {k}_{\mathrm{F}}^{-} $ represent the total concentration of the fluorescence dye Fluo-5N (0.1 mmol/L), and the association (48.8 (µmol/L)^−1^·s^−1^) and dissociation (19,520 s^−1^) constants in the reaction of Fluo-5N and Ca^2+^, respectively (Li *et al*. 2017).

Ca^2+^ flux through the RyR2 array is proportional to channel permeability and the concentration gradient between both sides of the channel. Thus,

6 $ {J}_{\mathrm{R}\mathrm{y}\mathrm{R}}=\frac{{i}_{\mathrm{R}\mathrm{y}\mathrm{R}}}{Far\times {V}_{0}}\times \sum ({\delta }_{i}\times \Delta {[{\mathrm{C}\mathrm{a}}^{2+}]}_{i}) , $

where *i*_RyR_ denotes the molar flux through a single open RyR2; *Far* is Faraday’s constant; *V*_*0*_ denotes the volume elements around RyR2; $ {\delta }_{i} $ indicates the state of the *i*th RyR2 channel with 1 as open and 0 as close; and $ \Delta {[{\mathrm{C}\mathrm{a}}^{2+}]}_{i} $ represents the gradient of [Ca^2*+*^] at the two sides of this opening RyR2, for which [Ca^2*+*^] at the cytosolic side is calculated simultaneously using a previously reported numerical model (Smith *et al*. 1998). The calcium current through a single opening RyR2 is ~0.6 pA upon a 1 mmol/L gradient of [Ca^2*+*^] (Mejia-Alvarez *et al*. 1999), thus, *i*_RyR_ = 0.6 pA.

SERCA is located in fSR; hence, the refilling flux of jSR contributes mostly via influx by connecting fSR tubules. Therefore,

7 $ {J}_{\mathrm{r}\mathrm{e}\mathrm{f}}={k}_{\mathrm{f}\mathrm{S}\mathrm{R}}\times \sum {([{\mathrm{C}\mathrm{a}}^{2+}]}_{\mathrm{f}\mathrm{S}\mathrm{R},i}-{[{\mathrm{C}\mathrm{a}}^{2+}]}_{i}) , $

where $ {[{\mathrm{C}\mathrm{a}}^{2+}]}_{\mathrm{f}\mathrm{S}\mathrm{R},i} $ and $ {[{\mathrm{C}\mathrm{a}}^{2+}]}_{i} $ represent Ca^2+^ concentrations inside and near the *i*th connecting fSR tubule, respectively; and constant *k*_fSR_ = 10 µm^2^/s denotes molar flux through single connecting fSR tubules (Brochet *et al*. 2005).

### Implementation of model

Our model is spatially detailed; that is, [Ca^2+^], [CaF], [CaCSQ], and all the fluxes are calculated with detailed spatial distribution. The jSR space is discretized into triangle grids through the Delaunay triangulation technique (Wu *et al*. 2009). Grid density is greater near fSR tubules and opening RyR2, where [Ca^2+^] gradient is larger. Equations 1–7 are numerically solved using the basic function method based on unstructured grids (Wu *et al*. 2009).

Initially, jSR space is under resting condition, and thus, intra-jSR [Ca^2+^] at every spatial grid node and [Ca^2+^]_fSR_ are 1.0 mmol/L, cytoplasmic [Ca^2+^] = 0.1 μmol/L, fluorescence compound [CaF] = 0.0714 μmol/L, and CSQ bound complex [CaCSQ] = 8.75 mmol/L.

The boundary of the computational domains is composed of the jSR circle and the circles of opening RyR2 channels. In accordance with Ca^2+^ movement at the boundary, it can be divided into three parts: the efflux boundary (RyR circle), influx boundary (sites of jSR that connect to fSR tubules), and wall boundary (jSR circle except influx boundaries).

In the influx and efflux boundaries, the fluxes *J*_ref_ and *J*_RyR_ should be calculated for the major control equation, *i*.*e*., Eq. 1, respectively. In the wall boundary, normal flux is prevented, *i*.*e*., $ \dfrac{\partial [{\mathrm{C}\mathrm{a}}^{2+}]}{\partial n}=0 $, where *n* denotes the normal vector of the wall boundary.

The program was encoded in Python language.

## Conflict of interest

Yutong Su, Yongshen Liang, Menghao Xu, Beibei Gao, Siyuan Zhang, Eric Yang, Shuai Yin, Da Li, Zhangqin Huang and Wenjun Xie declare that they have no conflict of interest.
